# Development and Clinical Application of a Novel Non-contact Early Airflow Limitation Screening System Using an Infrared Time-of-Flight Depth Image Sensor

**DOI:** 10.3389/fphys.2020.552942

**Published:** 2020-09-11

**Authors:** Hiroki Takamoto, Hiroki Nishine, Shohei Sato, Guanghao Sun, Sadao Watanabe, Kim Seokjin, Masahito Asai, Masamichi Mineshita, Takemi Matsui

**Affiliations:** ^1^Graduate School of Systems Design, Tokyo Metropolitan University, Tokyo, Japan; ^2^Department of Respiratory Medicine, St. Marianna University School of Medicine, Kanagawa, Japan; ^3^Japan Research Institute, Huawei Technologies Japan KK, Kanagawa, Japan; ^4^Graduate School of Informatics and Engineering, The University of Electro-Communications, Tokyo, Japan; ^5^Vital Laboratories, Pvt., Ltd., Tokyo, Japan

**Keywords:** early airflow limitation screening system, non-contact, ToF depth image sensor, home-use, FEV1, calibration free, COPD, asthma

## Abstract

Obstructive pulmonary diseases, such as diffuse panbronchiolitis (DPB), asthma, chronic obstructive pulmonary disease (COPD), and asthma COPD overlap syndrome (ACOS) trigger a severe reaction at some situations. Detecting early airflow limitation caused by diseases above is critical to stop the progression. Thus, there is a need for tools to enable self-screening of early airflow limitation at home. Here, we developed a novel non-contact early airflow limitation screening system (EAFL-SS) that does not require calibration to the individual by a spirometer. The system is based on an infrared time-of-flight (ToF) depth image sensor, which is integrated into several smartphones for photography focusing or augmented reality. The EAFL-SS comprised an 850 nm infrared ToF depth image sensor (224 × 171 pixels) and custom-built data processing algorithms to visualize anterior-thorax three-dimensional motions in real-time. Multiple linear regression analysis was used to determine the amount of air compulsorily exhaled after maximal inspiration (referred to as the forced vital capacity, FVC_*EAFL*__–SS_) from the ToF-derived anterior-thorax forced vital capacity (FVC), height, and body mass index as explanatory variables and spirometer-derived FVC as the objective variable. The non-contact measurement is automatically started when an examinee is sitting 35 cm away from the EAFL-SS. A clinical test was conducted with 32 COPD patients (27/5 M/F, 67–93 years) as typical airflow limitation cases recruited at St. Marianna University Hospital and 21 healthy volunteers (10/11 M/F, 23–79 years). The EAFL-SS was used to monitor the respiration of examinees during forced exhalation while sitting still, and a spirometer was used simultaneously as a reference. The forced expiratory volume in 1 s (FEV1%_*EAFL*__–SS_) was evaluated as a percentage of the FVC_*EAFL*__–SS_, where values less than 70% indicated suspected airflow limitation. Leave-one-out cross-validation analysis revealed that this system provided 81% sensitivity and 90% specificity. Further, the FEV1_*EAFL*__–SS_ values were closely correlated with that measured using a spirometer (*r* = 0.85, *p* < 0.0001). Hence, EAFL-SS appears promising for early airflow limitation screening at home.

## Introduction

Diffuse panbronchiolitis (DPB), asthma, chronic obstructive pulmonary disease (COPD), and asthma COPD overlap syndrome (ACOS), all known as typical obstructive pulmonary diseases, trigger a severe reaction including death for some situations (Laucho-Contreras et al., 2016). Spirometry is useful for early detection of these diseases with airflow limitation, although it is not designed for home-use. Early airflow limitation detections are critical to stop the disease progression. Here, we developed a novel home-use non-contact early airflow limitation screening system (EAFL-SS) using an infrared time-of-flight (ToF) depth image sensor (integrated into several smartphones) that does not require calibration to the individual by a spirometer.

Idiopathic inflammatory becomes a cause of DPB, which is frequently found in East Asia. DPB is a disease associated with respiratory bronchioles (Akaba et al., 2020). Respiratory bronchioles without treatment in early stage causes severe obstructive respiratory disorder, bronchiectasis, and death. Asthma is a syndrome of lung dysfunction involving airflow obstruction. The decrease of expiratory volume in 1 s (FEV1) is observed in patients with asthma. While, the number of deaths from COPD is increasing worldwide, and more than 90% of COPD-related deaths occur in low- and middle-income countries (World Health Organization [WHO], 2017). Patients with severe COPD require home oxygen therapy, which drastically degrades their quality of life. COPD is characterized by a persistent airflow limitation that is usually progressive. Early intervention, such as smoking cessation, it is particularly important to change the natural history of COPD. However, the noticeable symptoms (cough, sputum, and dyspnea) are not common in the early stages of COPD. Thus, early detection is crucial to effectively prevent the progression of COPD. In physiologically point of view, COPD is diagnosed by the FEV1/forced vital capacity (FVC) ratio (The Global Initiative for Chronic Obstructive Lung Disease [GOLD], 2020). As for COVID-19 hospitalized patients, COPD is the second risk factor (next to malignant tumor) of reaching to the composite end points (admission to Intensive Care Unit, invasive ventilation, or death) (Guan et al., 2020).

Spirometric measurements, especially the forced expiratory volume in 1 s evaluated as a percentage of the forced vital capacity (FEV1%), are the gold standard in airflow limitation screening and FEV1% values less than 70% indicate suspected airflow limitation. However, a conventional spirometer with a disposable mouthpiece can only be utilized for early airflow limitation screening with the help of a healthcare professional.

We have previously developed various medical screening systems, including respiratory measurement devices, such as a household-use Major Depressive Disorder screening system and infection screening systems, including a pediatric pneumonia monitor using a Doppler radar that determines a respiratory curve and estimates the respiration rate (Matsui et al., 2010; Sun et al., 2014, 2015, 2016; Dagdanpurev et al., 2018, 2019). A non-contact respiratory monitoring method using a fiber-grating vision sensor has been developed to evaluate the pulmonary functions of patients with airflow limitation (Tsujimura et al., 2011). This method successfully determined tidal volume with precision equivalent to that of a spirometer. However, this method requires several laser markers projected on the chest wall and related large-scale equipment. Soleimani et al. (2017) achieved accurate lung function measurement using a depth image sensor, but the proposed method required preliminary calibration using a spirometer for every new examinee.

**FIGURE 1:**
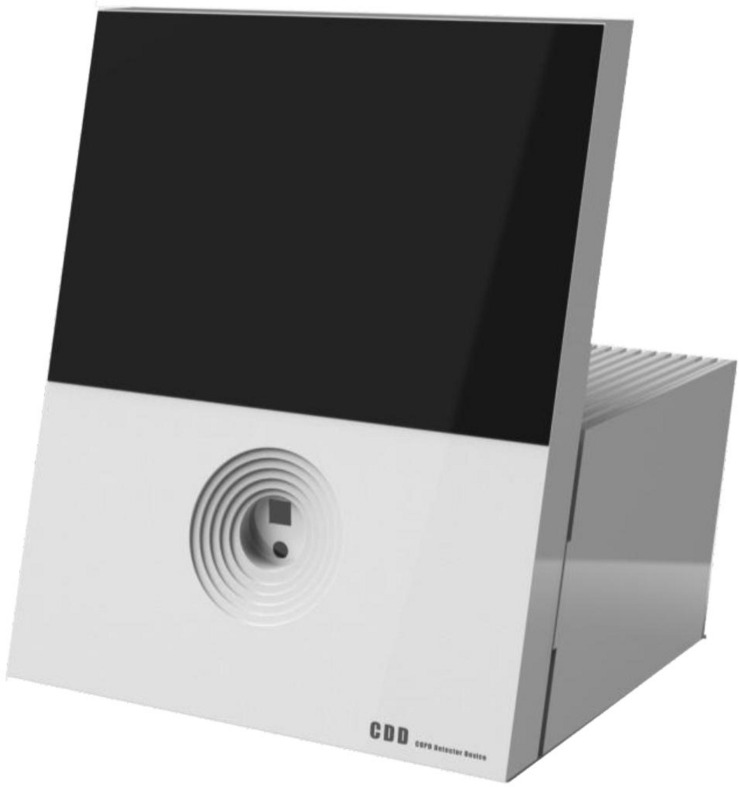
Exterior design of the early airflow limitation screening system (EAFL-SS), which includes an 850 nm infrared time-of-flight (ToF) depth image sensor for three-dimensional anterior-thorax motion measurement and an LCD display to provide instructions to the user.

To facilitate screening for airflow limitation at home without the help of healthcare professionals, we developed a novel non-contact EAFL-SS that does not require initial calibration. It relies only on an infrared ToF depth image sensor, which is integrated into several types of smartphones. The EAFL-SS monitors the three-dimensional (3D) motions of the anterior-thorax region of an examinee in real-time and derives a respiratory volume curve using multiple linear regression analysis (Figure 1). In contrast to the fiber-grating vision sensor, the ToF depth image sensor of the EAFL-SS is small (1.7 cm × 6.8 cm × 0.7 cm) and does not require any markers on the chest wall. Furthermore, unlike the system developed by Soleimani et al. (2017) the EAFL-SS does not require calibration to the individual using a spirometer. Instead, a multiple linear regression analysis was used to achieve early airflow limitation screening without calibration to the individual by deriving a volume curve from the 3D motion of the anterior thorax measured by the ToF depth image sensor and the examinee’s somatotypes [including height and body mass index (BMI)]. In addition, the distance from the EAFL-SS to the examinee was confirmed automatically to allow the accurate determination of a volume curve without calibration to the individual. A liquid crystal display (LCD) is used to direct the examinee to sit in an upright position, and the contact-free measurement is automatically started when an examinee is sitting 35 cm away from the EAFL-SS.

Here, we report the development and clinical testing of the EAFL-SS with 32 COPD patients as typical airflow limitation cases and 21 healthy subjects. The results were evaluated via leave-one-out cross-validation analysis (LOOCV).

## Materials and Methods

### Configuration of the EAFL-SS

An EAFL-SS prototype is shown in Figure 1. The prototype comprises a ToF depth image sensor, an 11-inch LCD, and a built-in personal computer. The ToF depth image sensor (CamBoard pico flexx, from PMD Technologies AG, Siegen, Germany), with built-in 850 nm infrared illumination of 38,304 (224 longitudinal × 171 lateral pixels), a viewing angle of 62° in the longitudinal direction by 45° in the lateral direction, and depth resolution of 2 mm) is mounted at the center of a funnel-like indent in the EAFL-SS and perpendicular to the ground (Figure 1). The ToF depth image sensor monitors the motion of the anterior thorax of an examinee in real-time by measuring the respective round-trip times of 850 nm infrared light reflected from the anterior-thorax region in each of the 38,304 pixels. The depth data determined by the infrared light flight time at each pixel is then transferred to the built-in personal computer via USB3.0 at a frame rate of 45 Hz.

### Data Processing Algorithms of the EAFL-SS

**FIGURE 2:**
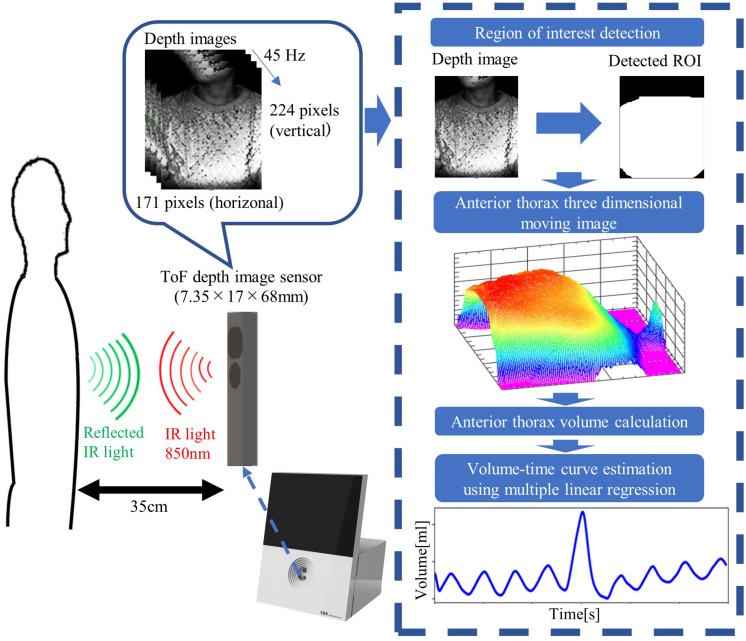
Data processing algorithms incorporated in the EAFL-SS. The system automatically creates a three-dimensional moving image of the examinee’s anterior thorax to derive a respiratory curve corresponding to the extracted anterior-thorax region of interest (ROI).

The data processing algorithm of the EAFL-SS is outlined in Figure 2. The EAFL-SS instructs the examinee via the LCD to sit 35 cm away from the device. The EAFL-SS automatically begins capturing 3D depth images of the examinee’s anterior thorax when the ToF depth image sensor senses that the examinee is in the correct sitting position. An automatic chest bounding box detection algorithm (Ostadabbas et al., 2016) was used to extract the thorax region in the captured images (Figure 2). The anterior-thorax depth image was binarized to eliminate background and other points more than 15 cm away from the initial sitting position. Then, the thorax region in the captured images was determined by removing the non-thorax region (i.e., neck and head) using this algorithm. The sampling and analysis software was written using LabView, a graphical block diagram programming language (National Instruments, Austin, TX, United States).

### Non-contact Volume Curve Evaluation Using a ToF Depth Image Sensor

**FIGURE 3:**
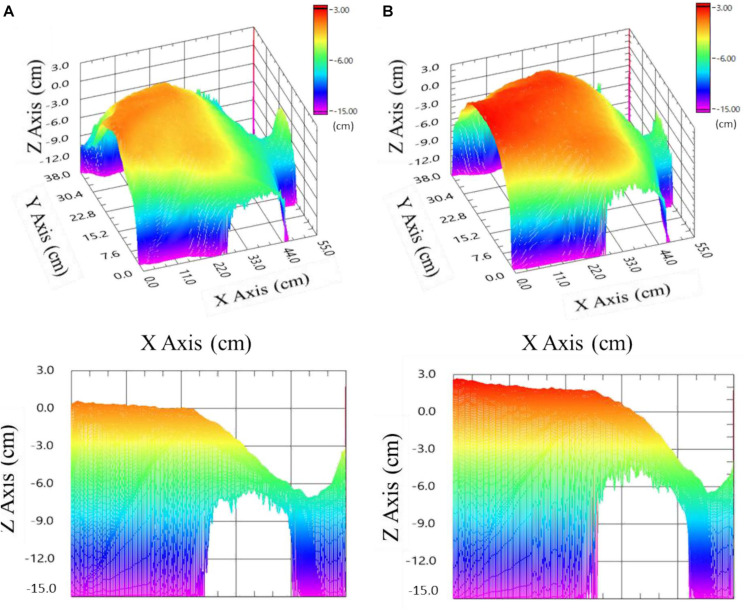
Images of the anterior thorax with neck and head (right part) of an examinee 0 cm on the *z*-axis corresponds to the examinee’s initial sitting position (35 cm away from the EAFL-SS). **(A)** Upper left: three-dimensional image at maximum exhalation. Lower left: sectional view at maximum exhalation. **(B)** Upper right: three-dimensional image at maximum inhalation. Lower right: sectional view at maximum inhalation.

The EAFL-SS measures the 3D movement of the anterior thorax using a ToF depth image sensor (Figures 2, 3). Because the depth image sensor of the EAFL-SS is mounted in front of an examinee, it cannot measure the posterior-thorax motions. Hence, to estimate the forced vital capacity (FVC_*EAFL*__–SS)_ using a single ToF depth image sensor, multiple linear regression analysis was conducted to determine FVC_*EAFL*__–SS_ from the FVC_*anterior–thorax*_ derived from the ToF depth image sensor, somatotype data (i.e., height and BMI) as explanatory variables and the spirometer-derived FVC_*spirometer*_ as the objective variable:

(1) $${\text{FVC}}_{\mathrm{EAFL}-\mathrm{SS}}=a\cdot {\text{FVC}}_{\mathrm{anterior}\,\mathrm{thorax}}+b\cdot \mathrm{Height}+c\cdot \mathrm{BMI}+d$$

**FIGURE 4:**
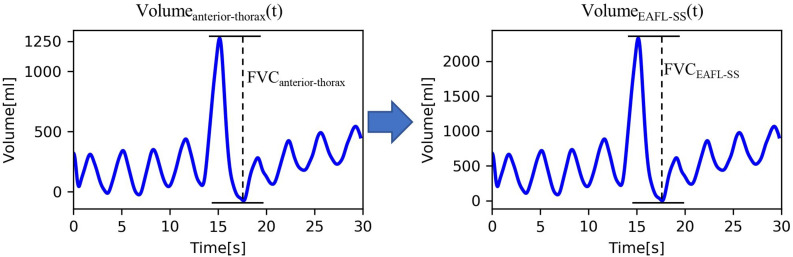
Conversion of ToF-derived anterior-thorax volume curve to EAFL-SS volume curve. FVC_COPD–SS_ = 0.55 × FVC_anterior thorax_ + 46.41 × Height + 0.29 × BMI − 5690.56.

Thus, the EAFL-SS-derived volume curve, Volume(*t*)_*EAFL*__–SS_, was determined by multiplying the anterior-thorax volume curve by the FVC_*EAFL*__–SS_/FVC_*anterior–thorax*_ ratio (Figure 4). Then, LOOCV analysis was used to evaluate the EAFL-SS performance without initial calibration.

### 3D Anterior-Thorax Image Reconstruction and FVC_anterior–thorax_ Determination

The area of each pixel is determined by its distance from the EAFL-SS. Thus, the 3D anterior image was reconstructed after area correction of each pixel using the following equation:

$$S_{\mathrm{ij}}=\left(2d_{\mathrm{ij}}\tan\frac{62{}^{\circ }}{2}\right)/224\left(\mathrm{vertical}\right)\times$$

(2) $$\left(2\,d_{\mathrm{ij}}\,\tan\,\frac{45{}^{\circ }}{2}\right)/171\,\left(\mathrm{horizontal}\right)$$

where S_*ij*_ is the pixel area of the ToF depth image sensor (1 ≤ *i* ≤ 224, 1 ≤ *j* ≤ 171), and d_*ij*_ is the distance between the EAFL-SS and the thorax surface corresponding to each pixel; 62° and 45° are the vertical and horizontal solid angles (transverse area at a distance), respectively. A Gaussian filter (7 × 7) was adopted to remove the high-frequency components of artifacts. The 3D anterior-thorax image shows alteration, which is synchronous with the timing of exhalation (Figure 3A) and inhalation (Figure 3B).

(3) $$\begin{array}{c} {\text{Volume}}_{\mathrm{anterior}-\mathrm{thorax}}\,\left(t\right)=\int_{0}^{t}\sum_{i=1}^{224}\sum_{j=1}^{171}\left({\text{S}}_{i\,j}\,\text{×}\,\Delta \,{\text{Z}}_{i\,j}\times {\mathrm{ROI}}_{i\,j}\right)\,\mathrm{dt} \end{array}$$

where ΔZ_*ij*_(*t*) is the change in distance from the ToF depth image sensor for a specific pixel (*i*, *j*) during a sampling duration of 22.2 ms (i.e., one sample at a sampling rate of 45 Hz), and ROI*_*ij*_* is 1 when S*_*ij*_* is included within the ROI and 0 when S*_*ij*_* is beyond the ROI.

The Volume_*anterior*−*thorax*_(*t*) of a healthy examinee is shown in Figure 4. FVC_*anterior–thorax*_ (eq. 1) corresponding to effort respiration is determined from the peak-to-peak amplitude of the Volume_*anterior*−*thorax*_(*t*).

### Respiratory Indices Evaluation

Each examinee was requested to make an effort to inhale and exhale without moving his or her body. The EAFL-SS can measure anterior-thorax movement through light clothing, such as T-shirt, polo shirt and blouse, but participants were asked to remove any thick clothing (such as coats or sweaters). Using the EAFL-SS and a spirometer (CHESTGRAPH HI-105, CHEST M.I., INC., Tokyo, Japan) as a reference simultaneously, the following respiratory parameters were determined: the forced expiratory volume in 1 s (FEV1_*EAFL*__–SS_, FEV1_*spiriometer*_), which is an important parameter to determine the severity of airflow limitation, the amount of compulsorily exhaled air amount after maximal inspiration, (FVC_*EAFL*__–SS_, FVC _*spirometer*_), which is referred as the FVC, and the FEV1 value as a percentage of the FVC value (FEV1.0%_*EAFL*__–SS_, FEV1.0%_*spirometer*_), which is an indicator of airflow limitation if it is less than 70%. To evaluate these respiratory indices without initial calibration, LOOCV analysis was conducted.

### Participants of the Clinical Test

A total of 32 COPD patients (27 men and 5 women, aged (67–93 years) as typical airflow limitation cases were recruited from the outpatient unit of the St. Marianna University School of Medicine Hospital. Additionally, 21 healthy volunteers (10 men and 11 women, aged 23–79 years) were recruited from the St. Marianna University School of Medicine Hospital and Tokyo Metropolitan University. All participants provided written informed consent.

### Measurement Protocol

The height and BMI of each examinee were recorded. Measurements for patients and healthy volunteers were conducted by a pulmonologist based on the ATS/ERS guideline. make were requested to sit on a chair 35 cm away from the EAFL-SS. The EAFL-SS automatically initiated the measurement when the examinee sat down 35 cm away from the sensor. After three normal respirations, the examinee was asked to make an effort to inhale and exhale without moving his or her body. The measurement time was 30 s. A spirometer as a reference was used simultaneously at the same time as EAFL-SS measurement. All measurements were conducted in sitting position.

## Results

### The Equation for FVC _*EAFL–SS*_ Determination

Multiple linear regression analysis yielded the following equation for determining the FVC _*EAFL*__–SS_:

$${\text{FVC}}_{\mathrm{EAFL}-\mathrm{SS}}=0.55\times {\text{FVC}}_{\mathrm{anterior}\,\mathrm{thorax}}+46.41\times$$

(4) Height+ 0.29×BMI- 5690.56

The EAFL-SS-derived volume curve, Volume _*EAFL*__–SS_(*t*), was determined by multiplying the *y*-axis of anterior-thorax volume curve by the ratio FVC _*EAFL*__–SS__/_FVC _*anterior–thorax*_ (Figure 4).

**FIGURE 5:**
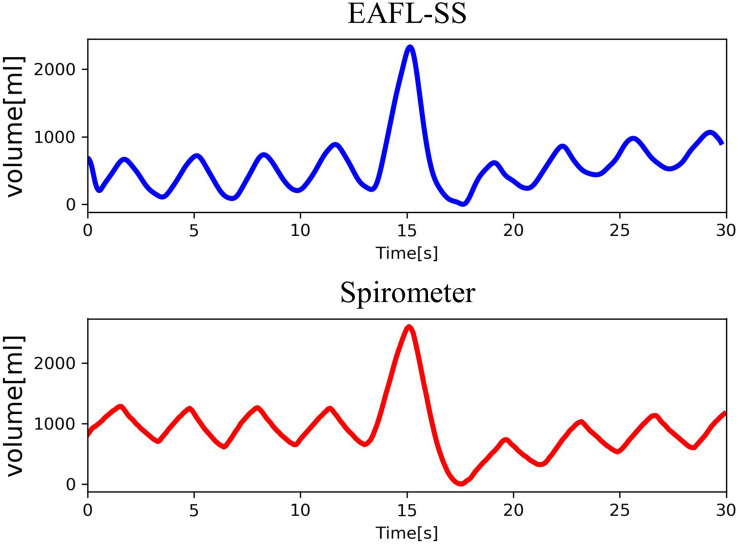
Comparison of volume curves derived from EAFL-SS **(top)** and a spirometer **(bottom)**.

Volume _*EAFL*__–SS_(*t*) and the respiratory curve measured by the spirometer, Volume _*spirometer*_(*t*), as a reference are shown in Figure 5. The changes observed in the non-contact-derived volume curve (Volume _*EAFL*__–SS_(*t*)) were similar to those in the contact-derived volume curve (Volume _*spirometer*_(*t*)).

### LOOCV Analysis to Determine Parameters Without Using One’s Own Spirometry Result

To enhance the utility of the EAFL-SS, it needs to be effective without preliminary calibration using a spirometer before every measurement. Therefore, we adopted LOOCV analysis to determine the FEV1_*EAFL*__–SS_, FVC _*EAFL*__–SS_, and FEV1.0%_*EAFL*__–SS_ of each examinee without using his or her spirometry results. LOOCV analysis was previously used in our small-scale study to evaluate a respiratory-related infection screening system (Yao et al., 2016).

### FEV1_*EAFL–SS*_ Evaluation for Airflow Limitation Severity

**FIGURE 6:**
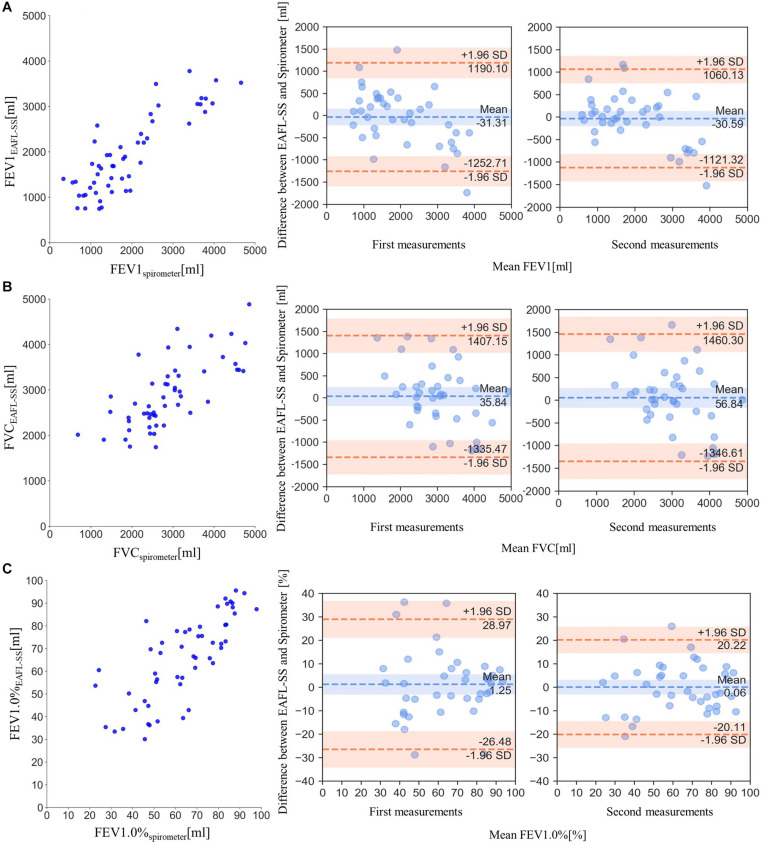
Comparisons of EAFL-SS-derived FEV1, FVC, and FEV1.0% with those obtained by spirometer, and the comparisons of Bland and Altman plots (FEV1, FVC and FEV1.0%) between first and second measurements to verify the repeatability of measurement. **(A)** Comparison of forced expiratory volume in 1 s (FEV1) derived from the EAFL-SS (FEV1_*EAFL*__–SS_) and a spirometer (FEV1_*spirometer*_). The comparison of Bland and Altman plot (FEV1) between first and second measurements. **(B)** Comparison of FVC values derived from the EAFL-SS (FVC_*EAFL*__–SS_) and a spirometer (FVC_*spirometer*_). The comparison of Bland and Altman plot (FVC) between first and second measurements. **(C)** Comparison of forced expiratory volume percentage in 1 s, (FEV1)/FVC × 100 (FEV1.0%), derived from the EAFL-SS (FEV1.0%_*EAFL*__–SS_) and a spirometer (FEV1.0%_*spirometer*_). The comparison of Bland and Altman plot (FEV1.0%) between first and second measurements.

FEV1_*EAFL*__–SS_, the index to determine airflow limitation severity, and FVC _*EAFL*__–SS_ were significantly correlated with FEV1_*spirometer*_ (Figure 6A) and FVC _*spirometer*_ (Figure 6B), respectively (FEV1_*EAFL*__–SS_: *r* = 0.85, *p* < 0.0001; FVC _*EAFL*__–SS__:_ *r* = 0.72, *p* < 0.0001).

FEV1.0% _*EAFL*__–SS_ can be used to determine airflow limitation: airflow limitation is suspected when FEV1.0%_*EAFL*__–SS_ is less than 70%. FEV1.0%_*EAFL*__–SS_ was significantly correlated with FEV1.0% _*spirometer*_ (*r* = 0.77, *p* < 0.0001), as shown in Figure 6C). The similarities of Bland and Altman plots (FEV1, FVC, and FEV1.0%) between first and second measurements revealed the repeatability of measurements (Figures 6A–C). Features of patients with airflow limitation and healthy volunteers are shown in Table 1.

**TABLE 1:** Comparisons between healthy volunteer and patients with airflow limitation (AFL).

	**Healthy volunteers (*n* = 21)**	**AFL patients (*n* = 32)**
Height, cm	163.8 ± 10.0	161.8 ± 8.5
Body weight, kg	57.0 ± 8.7	60.1 ± 14.3
VC, L	3.5 ± 0.9	2.5 ± 0.7
VC, % predicted	94.0 ± 10.4	78.3 ± 17.3
FEV1, L	2.9 ± 0.9	1.3 ± 0.5
FEV1, % predicted	94.2 ± 12.4	54.0 ± 20.1
FEV1/FVC, %	81.5 ± 9.4	54.9 ± 15.0

*AFL, Airflow Limitation; VC, vital capacity; FVC, forced vital capacity; FEV1, forced expiratory volume in 1 s.*

**FIGURE 7:**
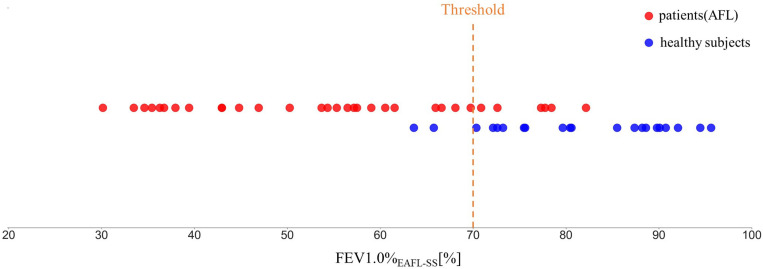
FEV1.0%_*EAFL*__–SS_ values observed in patients with airflow limitation and healthy volunteers (EAFL-SS determines an examinee to have airflow limitation when FEV1.0%_*EAFL*__–SS_ is less than 70%).

**FIGURE 8:**
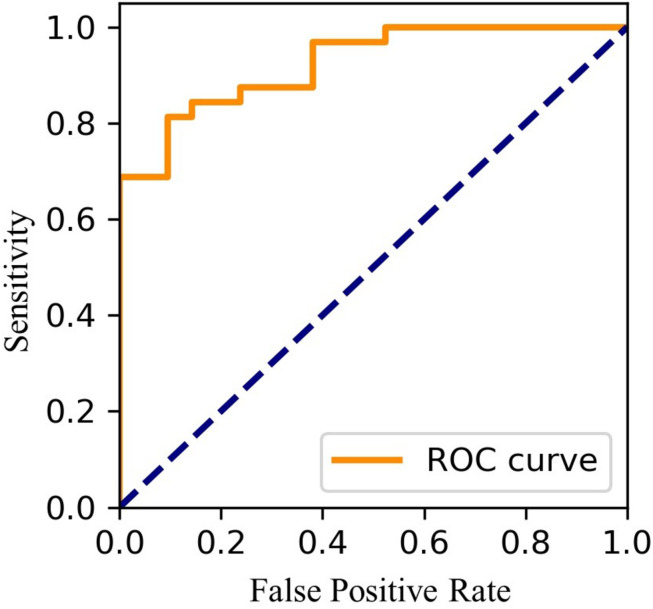
Receiver operating characteristic (ROC) curve.

Using FEV1.0%_*EAFL*__–SS_, EAFL-SS achieved 81% sensitivity and 90% specificity (Figure 7). Receiver Operating Characteristic (ROC) curve is shown in Figure 8.

## Discussion

### Effectiveness in Early Airflow Limitation Screening

Early airflow limitation screening system achieved 81% sensitivity and 90% specificity in airflow limitation screening. The proposed system succeeded to measure FEV1% (FEV1/FVC) and FEV1 without touching examinees using only ToF depth image sensor without conducting preliminary calibration via a spirometer. FEV1% and FEV1 are two dominant parameters to determine airflow limitations induced by asthma, COPD, and ACOS. “FEV1% < 70%” is an indicator of asthma, although it may improve spontaneously or by treatment. This value is also required for COPD diagnosis and is usually presented in ACOS patients. “FEV1 < 80% predicted” is compatible with asthma diagnosis and is an indicator of severity and future events including death for COPD and ACOS patients. The predicted values for FEV1 are calculated from age, race, height, and gender for healthy people (The Global Initiative for Chronic Obstructive Lung Disease [GOLD], 2015).

Chronic obstructive pulmonary disease, one of obstructive pulmonary disease, which is frequently induced by chronic smoking, is the third leading cause of death in the world (World Health Organization [WHO], 2017). It is important to screen for COPD to diagnose it at an early stage and to recommend changes in daily habits to COPD patients. COPD treatment is mainly treated only after it becomes symptomatic, but a standard therapy has not been established. Therefore, early airflow limitation screening is important to inform recommendations to people with the disease to improve their daily habits. EAFL-SS appears promising for early at-home detection of airflow limitation induced by early stage mild COPD but not severe COPD.

### Airflow Limitation Screening Without Initial Calibration

Soleimani et al. (2017) achieved accurate FEV1 and FVC measurements using a depth image sensor. However, that method required preliminary calibration using a spirometer for each new examinee. In contrast, the EAFL-SS successfully estimated the FEV1 and FVC without calibration by using an equation derived from multiple linear regression analysis with explanatory variables (i.e., combinations of somatotype parameters (height and BMI) and FVC _*anterior–thorax*_ derived from the 3D thorax ToF motion image). LOOCV analysis revealed that the EAFL-SS can be used to accurately determine the FEV1 and FVC without initial calibration using a spirometer.

In recent years, ToF depth image sensors have been built into several types of smartphones. Therefore, a smartphone loaded with the EAFL-SS data processing algorithm can be used as a portable EAFL-SS for personal use at home, for home care, and during sick visits.

### The Possibility in Unilateral Lung Obstruction Monitoring

Early airflow limitation screening system automatically creates a 3D anterior-thorax motion image (Figure 3). One of the co-authors of this study, Mineshita, reported that unilateral bronchial obstruction can be diagnosed and assessed based on the asynchrony in airflow between the left and right lungs, which is induced by a unilateral obstruction (2014). Thus, EAFL-SS has the potential to be used to diagnose unilateral lung obstruction by creating 3D asymmetric left and right thorax motion images. A conventional spirometer cannot be used for this purpose (Mineshita et al., 2014).

### Limitation of This Study

A limitation of the present study is the small sample size of patients and healthy volunteers. Therefore, future studies should involve more participants to further validate the accuracy of the FEV1-derived airflow limitation severity screening.

The preliminary results of this study indicate our system to be a promising device for early airflow limitation screening either at home or in the workplace without the need for initial calibration.

## Data Availability Statement

All datasets presented in this study are included in the article/Supplementary Material.

## Ethics Statement

The study was approved by Tokyo Metropolitan University Hino Campus Ethics Committee (No. H-19-007). The Research Ethics Committee at St. Marianna University School of Medicine approved this study (No. 3895). The patients/participants provided their written informed consent to participate in this study.

## Author Contributions

HT and TM designed the research and wrote the manuscript. KS and SW contributed to exterior design and development of system. HN and MM supervised the medical aspects of respiratory measurements and performed clinical testing. GS, SS, and MA contributed to the signal processing and image processing methods. All authors reviewed the manuscript.

## Conflict of Interest

SS is employed by company, Huawei Technologies Japan KK, and the research had received funding from this company, however it was not involved in the study design, collection, analysis and interpretation of data, the writing of this article nor the decision to submit it for publication. SW is employed by company, Vital Laboratories, Pvt., Ltd. The remaining authors declare that the research was conducted in the absence of any commercial or financial relationships that could be construed as a potential conflict of interest.

## Supplementary Material

VIDEO S1 Anterior-thorax three dimensional moving image of a healthy volunteer captured by infrared time of flight (ToF) depth image sensor.

VIDEO S2 The flow of data processing. Depth image ⇒ Anterior-thorax three dimensional moving image ⇒ Respiration curve corresponding to anterior-thorax.
